# Seroprevalence of Mumps among Patients Attending Selected Hospitals in Kenya

**DOI:** 10.24248/eahrj.v9i2.856

**Published:** 2025-12-24

**Authors:** Paul Muchai, Rosemary Nzunza, Joanne Hassan, Samoel Khamadi, Raphael Lihana, Vivienne Matiru, Peter Borus

**Affiliations:** a Centre for Virus Research, Kenya Medical Research Institute, Nairobi, Kenya; b Jomo Kenyatta University of Agriculture and Technology, Nairobi, Kenya

## Abstract

**Background:**

Mumps is a highly infectious viral disease that poses a significant public health concern despite presence of safe and effective vaccine. This study aimed to determine seroprevalence of mumps among patients attending selected hospitals in Coast and Central regions of Kenya, and to evaluate the demographic factors associated with mumps seropositivity.

**Methodology:**

This was a cross-sectional study where serum samples collected from patients attending Kiambu, Murang’a and Kilifi County hospitals were quantitatively tested for anti-mumps IgG antibodies using commercial enzyme linked immunosorbent assay (ELISA) kits. Sample size estimation was calculated using Cochran formula. A total of 451 volunteers were enrolled between October 2020 and November 2021. Ethical approval was obtained from Institutional Review Board of Kenya Medical Research Institute Scientific and Ethics Review Unit. Statistical analyses were conducted using Statistical Package for the Social Sciences (SPSS) version 23.0 software.

**Results:**

The overall mumps seropositivity rate was 90.5% and geometric mean of mumps IgG titers (GMT) was 401.3 U/ml. Both seroprevalence and GMT were not significantly influenced by the demographic factors of the population under study. Nonetheless, there were trends of higher prevalence related to certain demographic groups. Seroprevalence increased with increasing age from 85% for less than 10-year-olds to 100% among those aged between 60 and 69 years (*P*=.779). Similarly, females had a higher prevalence at 91.5% compared to 88.6% among males (*P*=.063). Also, the population from the central region of Kenya had a seroprevalence of 92% compared to 87.3% at the coast region (*P*=0.249) and rural residents reported 91.3% compared to 88.5% in urban residents (*P*=.607).

**Conclusions:**

The high mumps seroprevalence in this Kenyan population reflects widespread natural infection in the absence of vaccination. Age-related increases in antibody prevalence and geometric mean titres indicate cumulative exposure over time. These findings provide essential pre-vaccine baseline data and support the need for strengthened mumps surveillance and consideration of mumps vaccine inclusion in Kenya's national immunization program.

## BACKGROUND

Mumps is a contagious viral disease caused by the mumps virus, a member of the Paramyxoviridae family. It typically presents with fever, headache, and parotitis (swelling of the parotid glands), though complications such as orchitis, meningitis, and hearing loss can occur, particularly in adolescents and adults.^12^

Globally, the widespread use of the mumps-containing vaccine, especially as part of the measles-mumps-rubella (MMR) vaccine, has dramatically reduced the incidence of mumps in many countries. Despite vaccine availability, mumps outbreaks continue to occur even in countries with high MMR coverage. This is attributed to factors such as waning immunity, vaccine failure, and population movement.^3^

Mumps occurs worldwide, with an estimation of 500,000 cases reported on average annually.^4^ Mumps is known to occur mostly in crowded populations and few risk factors have been associated with mumps infection including, exposure, travel, vaccination status, time of year, and compromised immunity.^5^ Most mumps infections have been reported to occur during winter and spring in temperate zone, no mumps seasonality has been reported in tropical regions.^6^

In recent years, Europe and North America have experienced periodic outbreaks, especially among adolescents and young adults in congregate settings such as universities and military camps.^7^

In the African region, mumps is not a notifiable disease in most countries, and data are sparse. However, sporadic outbreaks have been reported, and the true burden is likely underestimated due to limited surveillance systems.^8^

While mumps has been largely controlled in high-income countries using mumps-containing vaccines (MuCV), its epidemiological profile in Africa remains poorly documented due to the absence of routine surveillance, limited diagnostic capacity, and exclusion from most national immunization schedules.^2^

Seroprevalence studies provide the most consistent evidence of endemic mumps virus circulation across Africa. In Sudan, a cross-sectional study in Khartoum revealed that only 63.6% of children aged 5 to 15 years had protective mumps IgG antibodies, with seropositivity increasing with age, reaching 94.1% among healthcare workers. This indicates ongoing virus transmission in the absence of vaccine-derived immunity.^9^ neither mumps nor rubella vaccines are currently used and comprehensive data on the seroepidemiology of measles, mumps, and rubella (M.M.R Similarly, in rural Tanzania, it was found that 88.8% of school-aged children (5–13 years) were seropositive for mumps IgG, underscoring widespread natural infection in areas without vaccination programs.^10^

Laboratory-confirmed case surveillance is rare in Africa, with South Africa being among the few countries conducting passive lab-based surveillance. Between 2012 and 2017, over 7,400 acute mumps cases were recorded, predominantly in children under 10 years of age. Mumps IgM positivity rates ranged from 13% to 19% annually, with bimodal seasonal peaks in June and November.^11^

The exclusion of mumps from routine immunization schedules across most African countries remains a significant public health gap. MuCV is not included in the Expanded Programme on Immunization (EPI) in most African nations. This creates a population-level susceptibility, particularly in densely populated urban settings where transmission risk is elevated. Moreover, global travel and migration further increase the potential for mumps virus importation and outbreaks.^2^

In the absence of routine vaccination, surveillance, and genotyping, the true burden of mumps in Africa remains underestimated. While outbreaks and serosurveys provide insight into transmission dynamics, there is an urgent need to expand laboratory confirmation and consider policy revisions that incorporate MuCV into routine immunization schedules to close the population immunity gap.

In Kenya, the national immunization program does not currently include the MuCV. The routine childhood vaccination schedule offers the measles-rubella (MR) vaccine, but mumps prevention relies entirely on natural immunity or access to private vaccination services.^12^ This leaves a significant portion of the population susceptible to mumps, especially children and adolescents who have not acquired natural immunity.

The absence of mumps vaccination in the public health program creates an immunization gap, a vulnerable population cohort lacking protective antibodies. This is especially concerning in urban and peri-urban areas where crowding facilitates virus transmission.^13^ Additionally, with global travel and migration, there is increasing potential for mumps virus importation and localized outbreaks, further exposing the gap in Kenya's population immunity.

Given the limited epidemiological data on mumps in Kenya and the absence of a national vaccination policy for mumps, this seroprevalence study provides a valuable tool to assess population-level immunity. It helps determine the proportion of individuals with mumpsspecific IgG antibodies, thereby identifying susceptible groups and estimating the risk of future outbreaks.

Such data are essential to inform immunization policy decisions, including the potential inclusion of the mumps vaccine in the national schedule. Additionally, it allows policymakers to weigh the cost-effectiveness of mumps immunization against the disease burden and outbreak risk. Seroprevalence studies can also reveal regional variations in immunity, enabling targeted interventions in high-risk areas.^3^ In Kenya's context, where routine surveillance for mumps is limited, serological data fill a crucial evidence gap.

The main objective of this study was to determine the seroprevalence of mumps among patients attending selected hospitals in Coast and Central regions of Kenya, and to evaluate the demographic factors associated with mumps seropositivity, to generate evidence that can inform national immunization policy and guide potential introduction of mumps-containing vaccines.

## METHODOLOGY

### Study Design, Duration, Study Area and Study Population

This was a cross-sectional hospital-based seroprevalence study conducted between October 2020 and November 2021 in selected health facilities within the Coast (Kilifi County) and Central (Kiambu and Murang’a counties) regions of Kenya. These regions were purposively selected to represent differing socio-epidemiological contexts, including variation in population density, urbanization, and access to healthcare.

The study involved volunteers aged 2 months to 90 years attending selected hospitals for non-infectious conditions. The hospitals involved included county hospitals with established clinical laboratories capable of supporting specimen collection and storage. Volunteers were recruited from general outpatient departments (OPDs), maternal and child health (MCH) clinics, and adult/pediatric medical wards.

### Sample Size Estimation, Sampling Procedures, and Selection Criteria

The Cochran formula was used to calculate minimum sample size:

N = Z^2^PQ/d^2^,

Where, N = the minimum sample size
Z = Confidence level at 95% (1.96)
*P* = 0.5 (assumed seroprevalence due to lack of prior Kenyan data, maximizing sample size),
Q = 1-P
d (5% margin of error).

Thus, N= (1.96)^2^ x 0.5 x 0.5 / (0.05)^2^ = 384

This yielded a minimum sample size of 384 participants. To allow for subgroup analysis by region, age group, and sex, and accounting for non-response or sample loss, the final sample was increased to 451 participants.

We employed a consecutive sampling strategy; eligible patients were approached in order of arrival during the study period until the target sample size for each facility was reached.

Patients attending selected health facilities during the study period and provided informed consent (or parental/guardian consent for minors) were included in the study while patients with bleeding disorders (for safety in venipuncture) and those who failed to provide consent were excluded from the study.

### Data Collection, Sample Collection & Storage

A data collection tool was used to collect volunteer's sociodemographic information including other relevant information such as vaccination status and history of mumps exposure. Venous blood samples (3–5 mL) were collected aseptically from each volunteer and placed in well-labeled, plain vacutainer tubes. The samples were triple-packed in a cool box at 4 to 8°C then transported to Kenya Medical Research Institute (KEMRI) measles and rubella laboratory where sera were extracted and stored in 2ml cryovials at −20°C pending testing.

### Laboratory Assay and Quality Control

Mumps-specific IgG antibodies were detected using a commercially available quantitative indirect enzymelinked immunosorbent assay (ELISA) kit (*SERION ELISA classic anti-mumps virus IgG, Virion\Serion GmbH, lot number: EL0134)* which has been validated for use in epidemiological studies in similar low-resource and tropical settings.^14^ the outbreaks of mumps remain frequent in China. Here we aim to assess herd immunity level followed by one-dose mumps ingredient vaccine and to elucidate the genetic characteristics of mumps viruses circulating in the post vaccine era in Jiangsu province of China. The complete sequences of mumps virus small hydrophobic (SH The assay sensitivity and specificity, as reported by the manufacturer, were >99% and >98%, respectively.

All testing was conducted at the Measles and Rubella laboratory, KEMRI – Center for Virus Research, which participates in external quality assurance programs for measles and rubella serology and follows standardized laboratory protocols. To minimize bias and ensure objectivity, serum samples were de-identified and blinded to laboratory personnel prior to testing.

Each assay run included ready-to use positive and negative controls plus standards provided in the kit. For each test run, the control and standard sera were included independent of the number of microtiter test strips used, and the standard sera were tested in duplicate. Random 10% of the samples were retested for reproducibility; concordance was >98%. Internal quality control charts were maintained to track absorbance readings and plate performance. While inter-laboratory variability was not directly assessed in this single-center study, the laboratory's experience in serological assays and strict adherence to good laboratory practices (GLP) ensured assay reliability and reproducibility.

Results were expressed quantitatively as optical densities (ODs) measurements at 405 nm using *Thermo-Labsystems-Multiskan*® Microplate Reader. The antibody concentration (U/ml) was calculated using software (easy*ANALYZE***®**) from *SERION* and was then categorized as negative, equivocal, or positive using fixed cut-off values following international standards according to kits’ manufacturer instructions. A value greater than 100 U/ml was considered positive, and a value of less than 30 U/ml was considered negative. Serum samples with titers of between 30 U/ml and 100 U/ml were categorized as equivocal. Equivocal samples were retested; persistent equivocal results were reported as such.

### Data Management and Analysis

Data was entered and managed in *Epi-Info* version 3.5.4 software and exported into Statistical Package for the Social Sciences (SPSS) version 23.0 software for analysis. The population was described by summarizing demographic characteristics and clinical history into percentages for categorical variables and mean with standard deviation for age. The seroprevalence of mumps was analyzed and presented as a percentage with 95% confidence interval (CI). Also, the geometric mean of IgG titers (GMT) was summarized as a mean with standard deviation, and 95% CI was calculated. Seroprevalence of mumps and GMT were associated with demographic features of the study population. The seroprevalence as a percentage was summarized by age group, gender, region, and area of residence and tested using Pearson Chi-square test. GMT were log-transformed to approximate normal distribution. The Shapiro–Wilk test and Q–Q plots were used to assess normality, and Levene's test was applied to evaluate homogeneity of variances. One-way ANOVA was used to compare GMTs across groups. Where assumptions were not fully met, results were validated using non-parametric Kruskal–Wallis tests. Statistical significance was interpreted as a *P value* of less or equal to .05.

Multivariable analysis was not performed due to limited variables and unmeasured confounders, and this is acknowledged as a study limitation.

### Study Limitations

This hospital-based study may not fully represent the general population since it may be introducing potential selection bias. The cross-sectional design limits interpretation of temporal trends in mumps exposure. Vaccination history was self-reported and may have been affected by recall bias. Finally, the study was conducted in only two regions, which may not reflect national variability in mump transmission.

### Study Strength

This study represents the first mumps seroprevalence investigation conducted in Kenya, providing essential baseline data on population immunity in a setting without routine mumps vaccination. The use of standardized commercial ELISA kits, coupled with rigorous laboratory procedures and quality-control measures, enhances the accuracy and reliability of the findings. Additionally, the large sample size and inclusion of participants spanning a wide age range improve the generalizability of the results and enable meaningful assessment of immunity patterns across different age cohorts.

### Ethical Consideration

Ethical approval was obtained from Institutional Review Board of Kenya Medical Research Institute Scientific and Ethics Review Unit (SERU) -SSC protocol No. 2605 (Ref. KEMRI/RES/7/3/1). Permission to conduct the study was also obtained from participating hospitals. Written informed consent was obtained from adult participants aged 18 years and above, adolescents aged 14–17 years provided written assent in addition to their parents/guardians’ consent. Consent was provided by guardians of minors below 14 years.

### Future Prospects

Future work should include longitudinal serosurveys to monitor changes in mumps immunity over time, particularly if a mumps-containing vaccine is introduced into the national immunization schedule. Establishing molecular diagnostics and genotyping capacity is essential to track circulating mumps virus strains and understand transmission patterns. Integrating mumps into routine national surveillance would strengthen outbreak detection. Broader, population-based studies across multiple regions are also needed to generate nationally representative data to guide vaccine policy.

## RESULTS

### Demographic Characteristics of Study Volunteers

A total of 451 records for study volunteers were used in data analysis. The study population recruited was aged between 2 months to 90 years. The samples from each region were stratified in the following age in years: <10, 10–19, 20–29, 30–39, 40–49, 50–59, 60–69, >70. As shown in Table 1, the median age was 30 years (IQR 21 and 46 years). Majority (63%) were females and 66.7% were from central region. In addition, slightly over two-thirds (69.2%) lived in the rural setting.

**TABLE 1:** Demographic Characteristics of Study Participants

Variable	Frequency (%)
Age in years	34.0 (19.0)
Mean (SD)	
Median (IQR)	30.0 (21.0–46.0)
Min-Max	0.25 – 90.0
Age category	
<10	40 (8.9)
10–19	52 (11.6)
20–29	123 (27.3)
30–39	79 (17.6)
40–49	58 (12.9)
50–59	43 (9.6)
60–69	33 (7.3)
70+	22 (4.9)
Gender	
Female	284 (63.0)
Male	167 (37.0)
Region	
Coast	150 (33.3)
Central	301 (66.7)
Area of residence	
Rural	312 (69.2)
Urban	139 (30.8)

### Clinical Characteristics

Only 3.8% of study population had received vaccination against mumps and 23.3% had a previous contact with mumps patient. A third (33%) had a history of parotitis and fever.

### Seroprevalence of Mumps and GMT

As shown in Table 2, the seroprevalence of mumps was 90.5% with a 95% confidence interval of 87.8 to 93.3%. Equivocal results were observed in 1.8% (n=8) of samples, while 7.8% (n=35) were seronegative for mumps IgG antibodies. The GMT of IgG titers in the population was 401.3 U/ml with a 95% CI of 359.9 to 441.3 U/ml. GMTs were not calculated for seronegative and equivocal samples as they fell below the assay's defined positivity threshold.

**TABLE 2:** Seroprevalence of Mumps and GMT

Variable	Frequency (%)	95% CI
Mumps immunity results		
Positive	408 (90.5)	87.8, 93.3
Negative	35 (7.8)	5.3, 10.2
Equivocal	8 (1.8)	0.7, 3.1
Geometric mean of IgG titers (GMT)	401.3	359.9, 441.3

### Seroprevalence of Mumps and GMT by Demographic Features

**FIGURE 1:**
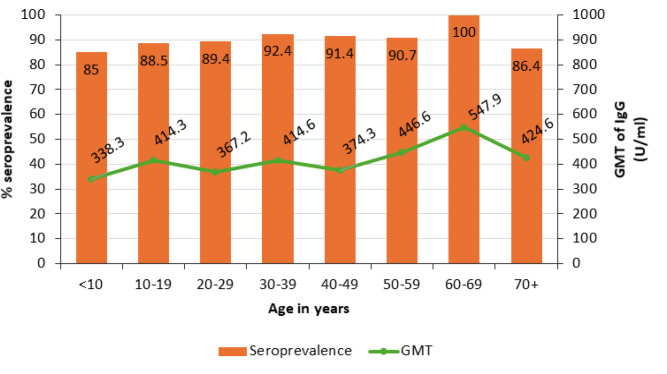
Seroprevalence and GMT by Age Group of Study Participants

As shown in Table 3, seroprevalence and geometric mean titers were not significantly influenced by the demographic factors of the population under study. However, there were trends of higher prevalence related to certain demographic groups. As shown in Figure 1, seroprevalence of mumps increased gradually with an increasing age group from 85% for less than 10-year-olds to 100% among those aged between 60 and 69 years (*P*=.779). Similarly, females had a higher prevalence at 91.5% compared to 88.6% among males (*P*=.063). Also, the population from the central region of Kenya had a seroprevalence of 92% compared to 87.3% at the coast region (*P*=.249) and rural residents reported 91.3% compared to 88.5% in urban residents (*P*=.607).

**TABLE 3:** Seroprevalence of Mumps and GMT by Demographic Features

Variable	Seroprevalence			GMT		
	Positive, n (%)	95% CI	*P value*	Mean	95% CI	*P value*
Age in years						
<10	34 (85.0)	72.5, 95.1	.779	338.3	242.3, 455.8	.591
10–19	46 (88.5)	79.5, 96.2		414.3	304.5, 550.6	
20–29	110 (89.4)	83.5, 94.5		367.2	298.0, 440.2	
30–39	73 (92.4)	86.4, 97.8		414.6	331.6, 517.0	
40–49	53 (91.4)	83.9, 98.1		374.3	271.3, 497.2	
50–59	39 (90.7)	81.4, 97.9		446.6	314.1, 607.6	
60–69	33 (100.0)	-		547.9	412.0, 737.7	
70+	19 (86.4)	70.0, 100.0		424.6	283.4, 651.4	
Gender						
Female	260 (91.5)	88.3, 94.5	.063	421.2	368.7, 475.6	.208
Male	148 (88.6)	83.4, 93.1		369.6	314.5, 433.5	
Region						
Coast	131 (87.3)	81.8, 92.5	.249	365.1	310.3, 429.1	.184
Central	277 (92.0)	89.0, 95.1		420.6	373.7, 477.5	
Area of residence						
Rural	285 (91.3)	88.1, 94.4	.607	408.6	364.1, 460.5	.589
Urban	123 (88.5)	82.9, 93.5		385.3	322.1, 461.1	

## DISCUSSION

This study found a high overall mumps IgG seroprevalence of 90.5% among patients attending selected health facilities in Kenya's Coast and Central regions. This is notable given that mumps vaccination is not included in Kenya's national immunization schedule. Only 3.8% of volunteers self-reported prior vaccination, strongly suggesting that the detected antibodies result from natural infection.

Equivocal and negative results were distributed across all demographic groups without significant clustering. Their presence underscores possible waning immunity, lack of vaccination, or delayed seroconversion, especially in the younger cohorts.

High seroprevalence in unvaccinated populations has similarly been observed in other low- and middle-income countries. For example, a 2020 study in Sudan, where mumps vaccination is also not routine, reported seroprevalence ranging from 63.6% in children to 97.1% in adults.^9^ Study conducted in In Lao-PDR, found a steady rise in seropositivity with age — from 10.6% in children aged 1 to 2 years to nearly 90% in adults.^15^ Our findings follow this trend: seroprevalence increased from 85% in those under 10 years to 100% among those aged 60–69 years, indicating cumulative exposure over time.

This age-associated increase in immunity is consistent with natural mumps virus circulation. Without vaccination, individuals acquire immunity through repeated environmental exposure. The geometric mean concentrations (GMCs) of mumps antibodies also increased with age, mirroring seroprevalence trends, and aligning with findings from unvaccinated populations in Germany and China.^16,17^

Regional comparison revealed slightly higher seroprevalence in the Central region (92%) than the Coast (87.3%), though the difference was not statistically significant (*P*=.249). Likewise, rural residents (91.3%) showed marginally higher seropositivity than urban residents (88.5%, *P* =.607). These differences may reflect variations in exposure intensity but are unlikely to be programmatic, as mumps vaccine is not offered in either setting. Previous studies in Turkey and China also found no consistent pattern in mumps seroprevalence based on rural versus urban residency in unvaccinated populations.^18,19^

Gender differences were also minor. Females had a higher seroprevalence (91.5%) than males (88.6%), though the difference was not significant (*P* =.063). This trend aligns with reports of slightly stronger humoral immune responses in females, ^20,21^ though such findings may be influenced by biological and hormonal factors that were not assessed in this study.

Importantly, this dataset provides crucial baseline information for Kenya, where the burden of mumps is poorly understood and routine surveillance is limited. Mathematical models estimate herd immunity thresholds for mumps at 85%–92%, ^17^ suggesting that the observed seroprevalence may be sufficient to limit widespread transmission in some age groups. However, such natural immunity carries risks, especially complications from mumps infections in adolescents and adults — and is not a substitute for structured, safe immunization.

While some studies have examined climatic influences on mumps incidence, ^19,22^ this study did not find significant regional differences attributable to environmental factors, and such associations remain speculative in our context. Further research using surveillance data would be needed to evaluate any ecological effects on virus transmission.

## CONCLUSION

This study provides initial data on mumps seroprevalence in Kenya, offering baseline information in a population without routine mumps vaccination. The high mumps seroprevalence in this Kenyan population reflects widespread natural infection in the absence of vaccination. Age-related increases in antibody prevalence and geometric mean titres indicate cumulative exposure over time. These findings support the need for strengthened mumps surveillance and consideration of mumps vaccine inclusion in Kenya's national immunization program to reduce susceptibility and prevent future outbreaks. In parallel, expanding laboratory capacity for molecular detection and genotyping is essential to improve case confirmation, monitor transmission, and guide evidence-based public health decision-making.
